# Synthesis of Ag/NiO Honeycomb Structured Nanoarrays as the Electrode Material for High Performance Asymmetric Supercapacitor Devices

**DOI:** 10.1038/s41598-019-41446-0

**Published:** 2019-03-19

**Authors:** Sadayappan Nagamuthu, Kwang-Sun Ryu

**Affiliations:** Department of Chemistry, University of Ulsan, Muger-dong, Nam-gu, Ulsan, 680-749 Republic of Korea

## Abstract

Metallic silver nickel oxide honeycomb nanoarrays were synthesized via a surfactant-assisted hydrothermal route. The crystal structure of the Ag/NiO nanoarrays was confirmed by X-ray diffraction. X-ray photoelectron spectroscopy confirmed the valance state of the nickel, oxygen, and metallic silver. The morphological studies and energy dispersive X-ray spectroscopy revealed the honeycomb structured nanoarrays and the elemental distribution of the prepared sample, respectively. The three-electrode measurements showed that the Ag/NiO nanoarray is a suitable electrode material for supercapacitor applications, which delivers the maximum specific capacity of 824 C g^−1^ at a specific current of 2.5 A g^−1^. An Ag/NiO positive electrode-based asymmetric device was fabricated and tested. The asymmetric device yielded a high specific cell capacity of 204 C g^−1^ at a specific current of 2.5 A g^−1^ as well as a maximum energy density of 63.75 W h kg^−1^ at a power density of 2812.5 W kg^−1^. These results are comparable to those of (NiMH) metal hydride batteries.

## Introduction

The worldwide electricity demand has increased continually over the past few decades due to rapid population growth and the increased use of electricity for a comfortable living. The fossil fuel reserves and their resource constraints highlight the need to generate electricity from renewable energy sources and develop low-cost electrical energy storage devices. Electrochemical energy storage devices are believed to be a key to charge storage devices, such as batteries, conventional capacitors, and supercapacitors. Supercapacitors provide high power density, long cycle life, and safe operation. One major problem with supercapacitors is their lower energy density in aqueous electrolytes^1^. The energy density of supercapacitors can be improved by the fabrication of asymmetric supercapacitor devices.

Carbon nanotubes (CNTs), graphene, activated carbon, conducting polymers, RuO, NiO, Co_3_O_4_, V_2_O_5_, and MnO_2_ have been used as the electrode materials for supercapacitors^2–10^. Carbon-based materials show limited specific capacitance and sustain a cycle life of millions of cycles. Transition metal oxides yield a high specific capacitance and good cycle life. Among these materials, nickel oxide is a suitable pseudocapacitance material owing to its environmentally friendly nature, high reversible redox reaction, and high theoretical capacitance (3750 F g^−1^ in the alkaline electrolyte).

The electrical conductivity of the electrode active material plays a vital role in electrochemical reactions, which boost the power performance of supercapacitors. Nickel oxide has the low electrical conductivity which restricts the power performance of the capacitor^11^. The electrical conductivity has been improved by the addition of graphene, CNT, acetylene black, and silver in NiO nanostructures electrodes^12–21^. Among these materials, silver increases the electrical conductivity and enhances the electrolyte ion diffusion and high utilization of the electroactive sites in the nickel oxide electrode. B. Wu *et al*.^22^ used a single source precursor for the Ag/NiO composite for supercapacitor applications and reported a capacitance of 1116 C g^−1^ at 2 A g^−1^. Y. Yang *et al*.^23^ reported dilute NiO/carbon nanofiber composites synthesized from the metal-organic framework. Dilute NiO/carbon nanofiber composites yielded 234 F g^−1^ at a scan rate of 1 mV s^−1^. N. Padmanathan *et al*.^24^ reported NiO-In_2_O_3_ micro flower (3d)/nanorod hetero-architecture as a supercapattery electrode with good cyclic stability. This hetero-architecture NiO nanocomposites electrode delivered a maximum specific capacity of 766.65 C g^−1^ at 5 A g^−1^. S. Xu *et al*.^25^ used nanofoaming to boost the electrochemical performance of the Ni@Ni(OH)_2_ nanowires for ultrahigh volumetric supercapacitor electrodes. They fabricated an asymmetric device (here they used Ni@Ni(OH)_2_ nanowires as the positive electrode and graphene-carbon nanotubes as the negative electrode) and estimated a volumetric capacitance of 50 F g^−1^ at 2 A cm^−3^.

This paper reports the single route synthesis of metallic silver nickel oxide honeycomb nanoarrays architecture and its electrochemical performance of the supercapacitor electrode. X-ray diffraction (XRD) revealed NiO mixed with metallic silver peaks. The binding energy of the metallic silver indicated the presence of silver in the prepared NiO honeycomb nanoarrays, which was analyzed by X-ray photoelectron spectroscopy (XPS). The three electrode measurements showed the Ag/NiO honeycomb nanoarrays are a suitable electrode material for hybrid supercapacitor applications. Finally, a hybrid supercapacitor device was fabricated using metallic silver with NiO honeycomb nanoarrays as the positive electrode and activated carbon as the negative electrode. The electrochemical performance of the hybrid supercapacitor device was tested.

## Methods

Silver nitrate, nickel (II) nitrate hexahydrate, hexamethylenetetramine (HMT), and urea were purchased from Sigma-Aldrich and used as the precursor sources. Nickel (II) nitrate hexahydrate (0.2617 g) and silver nitrate (0.15288 g) was dissolved separately in 30 ml of double distilled water. The above two solutions were mixed together and stirred continuously for 15 minutes. Subsequently, 100 mM of HMT (10 ml) was added to the precursor solution. A 0.6 M urea solution (20 ml) was added to the above solution and stirred for 30 minutes. The final solution was transferred to a Schott Duran glass bottle with a tightly closed polypropylene screw cap, which was kept at 100 °C in an oven for 24 h. The precipitate was collected by centrifugation and washed several times with water and ethanol. The collected samples were dried at 100 °C for 24 h and calcined at 400 °C for 2 hours at a heating rate of 5 °C/min. The calcined sample was used for further characterization and called Ag/NiO. XRD (Rigaku-Ultima 4) of the Ag/NiO honeycomb nanoarrays was performed using Cu Kα radiation. The valance distribution of the prepared Ag/NiO samples was examined by XPS (Thermo Scientific) using an Al K alpha X- ray source. FESEM (Jeol-JSM7600F) and HRTEM (Jeol-JEM-2100F) was used to analyze the surface morphology, energy-dispersive X-ray spectroscopy (EDAX), and elemental mapping. A nano POROSITY surface area analyzer (Miar Scientific Instruments Inc.) was used measure the N_2_ adsorption/desorption.

The pseudocapacitance of the Ag/NiO samples was examined using an IVAM- STAT instrument for the three and two electrode methods. The working electrode consisted of the active material, acetylene black, and polytetrafluoroethylene at a ratio of 85:10:5, which were pasted onto a nickel foam (1 cm × 1 cm and 1 mg active material) current collector. Platinum wire and an Ag/AgCl electrode were used as the counter and reference electrodes, respectively. A 2 M KOH solution was used as the electrolyte. The energy density and power density was estimated for the asymmetric device.

## Results and Discussions

### Structural and Morphological studies

**Figure 1:**
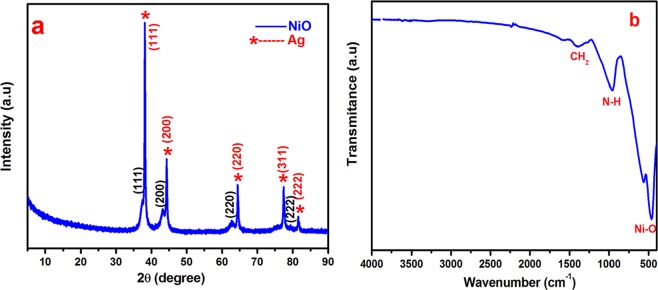
(**a**) XRD pattern and (**b**) FTIR spectra of Ag/NiO nanoarrays.

Figure 1(a) shows the XRD pattern of the Ag/NiO sample. The diffraction angles agreed well with the cubic crystal structure of nickel oxide with metallic silver. The peaks at 37.22°, 43.254°, 62.83°, and 75.35° 2θ corresponded to the JCPDS card No-73-1523 for NiO. The corresponding metallic silver peaks were observed at 38.26°, 44.47°, 64.71°, 77.74°, and 81.91° 2θ, which agreed with the JCPDS card No-87-0719. XRD showed that the prepared sample was metallic silver with nickel oxide. Silver enhances the electronic conductivity of the NiO nanoarrays, which is more favorable for the electrochemical redox reaction at the electrode/electrolyte interface.

FTIR spectrum was shown in Fig. 1(b) in the range of 400 to 4000 cm^−1^. The peaks appear at 475 and 568 cm-1in the spectrum which was attributed to the Ni-O stretching vibrations. N-H wagging mode and CH_2_ stretching modes was observed at 953 and 1422 cm^−1^ which may arise from surfactant (HMT)^26,27^. The optical study of Ag/NiO honeycomb nanoarrays was studied using UV-vis spectroscopy. The optical absorbance spectrum was presented in supporting information Figure S1. The spectrum indicates the two maximum absorbance bands at 340 and 552 nm which were confirmed that electronic transition from valance band to conduction band in the NiO nanoarrays semiconductor^28^.

**Figure 2:**
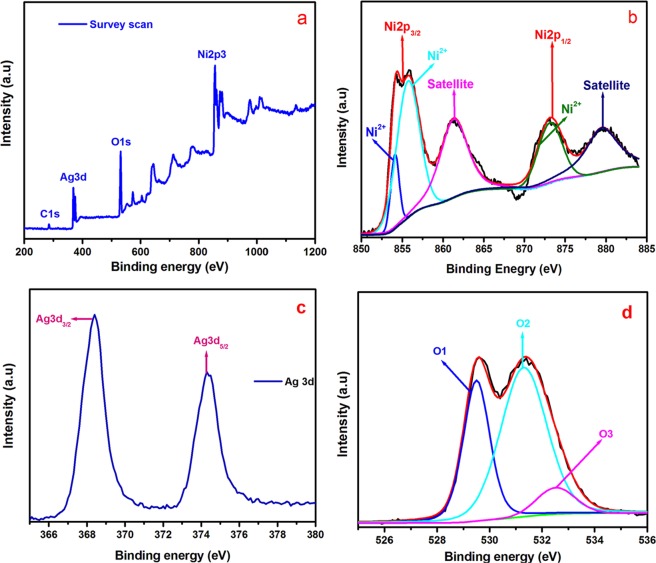
(**a**) Survey spectrum of Ag/NiO, (**b**) high resolution spectrum of Ni 2 P, (**c**) high resolution spectrum of Ag 3d, and (**d**) high resolution spectrum of O1s for Ag/NiO nanoarrays.

The valance state of nickel, silver, and oxygen was determined by XPS. Figure 2(a) presents the survey scan of the Ag/NiO honeycomb nanoarrays. The survey scan confirmed the presence of the material and its oxidation valance states (Ni2p, Ag3d, and O1s). Figure 2(b) shows the core level spectrum of Ni 2p (Ni^2+^), which is comprised of five distinct peaks. The binding energies at 854.1, 855.4 eV and 861.5 eV were assigned to the Ni2p_3/2_ state and satellite peak respectively. The binding energy at 854.1 eV (Ni^2+^) peak ascribed to the local screening from lattice oxygen to the Ni 2p core hole and 855.4 eV attributed to the nonlocal screening from the lattice even though which has been reported contain a partial contribution from surface states. The Ni2p_1/2_ state was observed at 874.1 eV which was arose from Ni-O species and its corresponding satellite peak was observed at 879.6 eV^29^. Figure 2(c) presents the high-resolution spectrum of Ag 3d, which displays two peaks at 368.3 and 374.3 eV. The former and latter peaks were assigned to the Ag 3d_3/2_ and Ag 3d_5/2_, respectively, which indicate the presence of metallic silver in the Ag/NiO honeycomb nanoarrays^30^. Figure 2(d) shows the high-resolution spectrum of oxygen. The major peaks were observed at the 529.4, 531.2 and 532.5 eV, which were assigned to lattice oxygen (O^2−^), adsorbed water and hydroxyl groups (OH)^31^. Both XRD and XPS confirmed the presence of nickel oxide and metallic silver in the prepared samples.

**Figure 3:**
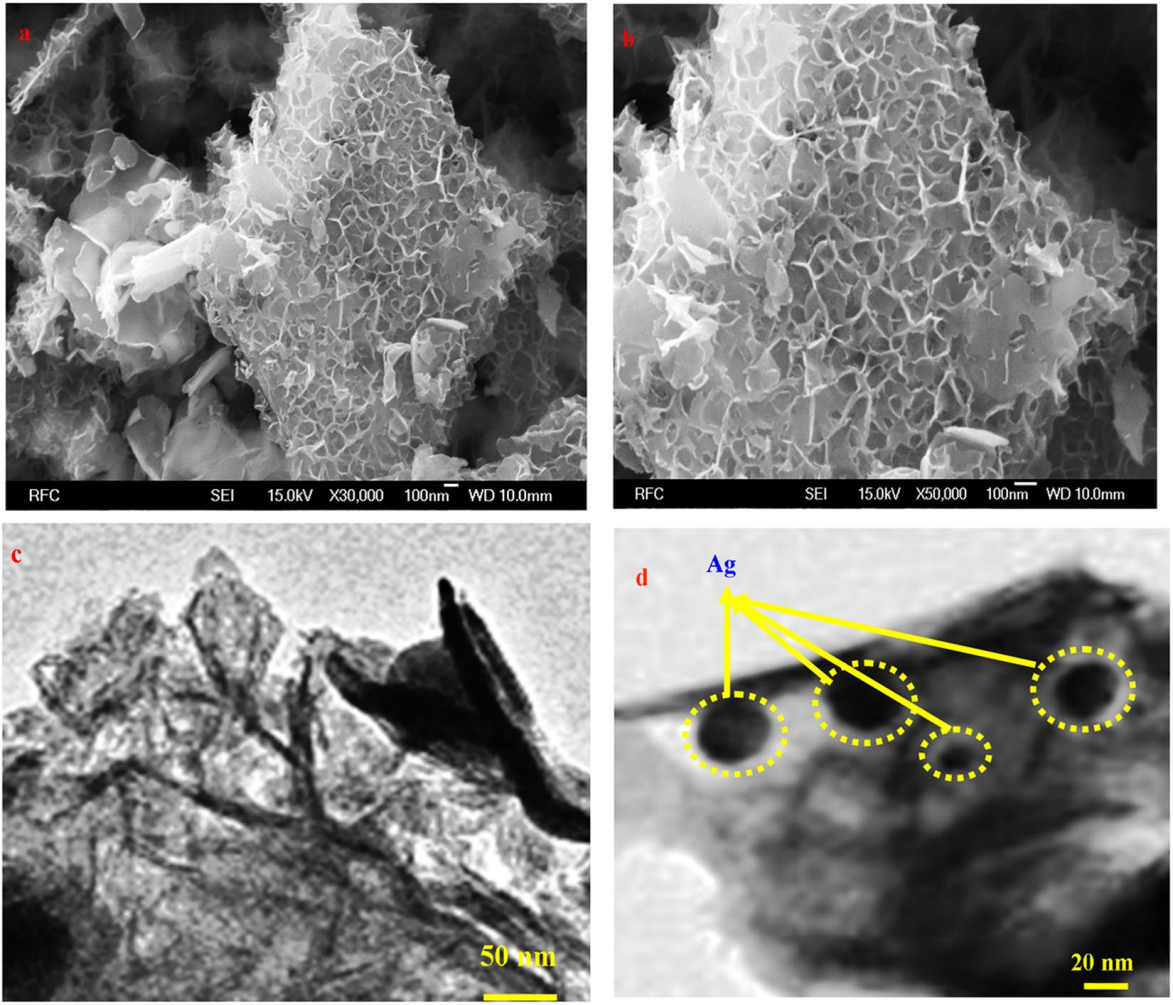
(**a** and **b**) FESEM images of the Ag/NiO nanoarrays and (**c** and **d**) HRTEM images of the nanoarrays at various magnifications.

**Figure 4:**
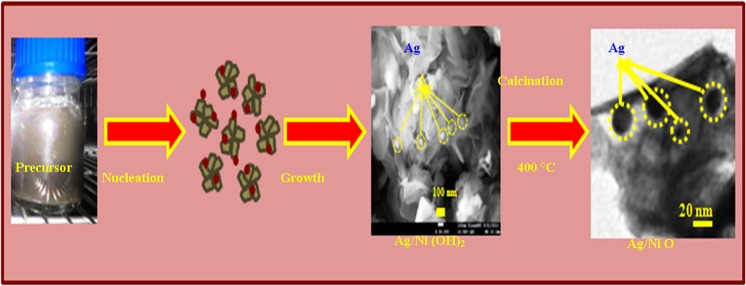
Proposed formation mechanism of Ag/NiO nanoarrays.

Figure 3(a and b) shows FESEM images of the metallic silver nickel oxide nanoarrays. Honeycomb structured nanoarrays consisting of thin Ag/NiO nanosheets were observed. Figure 3(c and d) presents HRTEM images of Ag/NiO nanoarrays. These images confirm the honeycomb nanoarray structures. This nanoarray has a high contact area, larger amount of active material in a unit area and high porosity at the electrode/electrolyte interface, which is more feasible for supercapacitor applications^32^. The formation of Ag/NiO honeycomb nanoarrays involves hydrolysis, nucleation, Ostwald-ripening mechanism, and precipitation (hydrothermal route). Figure 4 presents the proposed mechanism which has been explained through the following chemical reactions^33^.

1 $$\begin{array}{c}{{\rm{Ag}}}^{+}+{\rm{HCHO}}+{{\rm{NH}}}_{3}\to {\rm{Ag}}+\downarrow {{\rm{NH}}}_{4}^{+}+{{\rm{OH}}}^{-}\\ {{\rm{Ni}}}^{2+}+2{({\rm{OH}})}^{-}\to {\rm{Ni}}{({\rm{OH}})}_{2}\downarrow \\ {\rm{Ag}}+{\rm{Ni}}{({\rm{OH}})}_{2}\,\underset{400^\circ {\rm{C}}}{\overset{{\rm{\Delta }}}{\longrightarrow }}\,{\rm{Ag}}/{\rm{NiO}}\end{array}$$

The HMT decomposed into formaldehyde and ammonia under hydrothermal conditions and ammonia then reacted to form NH_4_^+^ and hydroxyl ions (OH^−^). Formaldehyde was used as the reducing agent, which reduces silver nitrate to metallic silver^34^. Here, urea acts as the hydrolysis agent and decomposes to form hydroxyl ions (OH^−^) in aqueous media and further reacts with Ni^2+^ to form Ni(OH)_2_. The OH^−^ ions supply was controlled by HMT, which controls the rate of hydrolysis and nucleation. Many nuclei disintegrate and grow further into particles and then to nanosheets. The primary nanosheets could assemble to a flower-like structure due to the crystal face attraction, van der Waals forces and hydrogen bonding under hydrothermal conditions. Heterogeneous nucleation occurred, which controlled the rate of dissolution/recrystallization, leading to the formation of a flower-like structure (Ostwald –ripening mechanism)^35^. After calcination, the flower-like structure transformed to the honeycomb nanoarrays.

**Figure 5:**
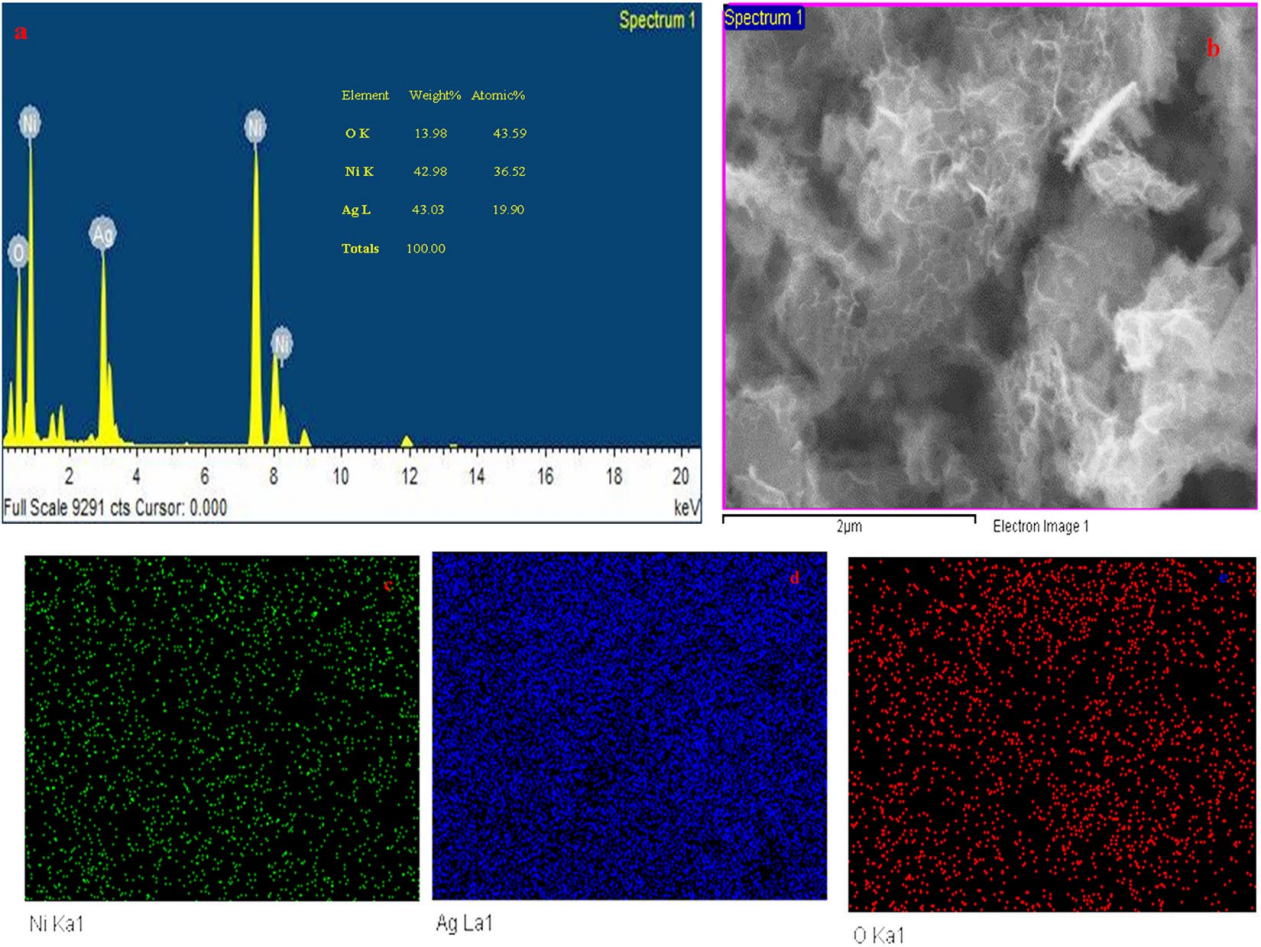
(**a**) EDAX spectrum of Ag/NiO, (**b**) Area of the EDAX measurement of Ag/NiO, (**c**) elemental mapping of Ni, (**d**) elemental mapping of Ag, and (**e**) elemental mapping of O for Ag/NiO nanoarrays.

Figure 5(a–e) presents the EDAX pattern of Ag/NiO honeycomb nanoarrays and elemental mapping of the corresponding EDAX pattern. The inset of the table demonstrates the elemental composition of the prepared samples. EDAX shows that the prepared samples contain silver, nickel, and oxygen. The metallic silver maintains the internal resistance of the electrode materials, which helps enhance the rate performance of the supercapacitor.

**Figure 6:**
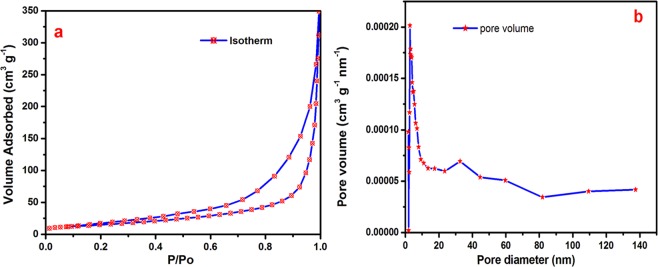
(**a**) N2 – adsorption/desorption curve and (**b**) pore size distribution curve of the Ag/NiO nanoarrays.

Figure 6(a and b) shows the nitrogen (N_2_) adsorption–desorption and pore size distribution of the metallic silver nickel oxide honeycomb nanoarrays. The hysteresis loop of the isotherm curve indicates a type IV isotherm curve according to the IUPAC classification. The hysteresis loop was associated with adsorption/desorption of the micropores (<2 nm) and mesopores (2–5 nm) by capillary condensation starting at P/Po = 0. 225 extending almost up to P/Po = 0.99. This highlights the high level of porosity in the Ag/NiO honeycomb nanoarrays. The pore size distribution curve illustrates the narrow pore size of the peak centered at 2.5 nm which was calculated from the N_2_ adsorption/desorption branches using the BJH method. The pore diameter of the electrode material is greater than 2 nm, which is more favorable for aqueous electrolytes. The Ag/NiO nanoarrays sample had a BET surface area of 54.01 m^2^ g^−1^ and a pore volume of 0.3769 cm^3^ g^−1^. The Ag/NiO honeycomb nanoarrays showed a higher porosity, narrower pore size distribution, higher BET surface area, and higher pore volume than that reported elsewhere^36,37^, which offers effective electron transport at the electrode/electrolyte interface.

### Electrochemical studies

The electrochemical performance of the metallic silver/nickel oxide honeycomb nanoarrays was measured by cyclic voltammetry (CV), galvanostatic charge-discharge, and ac-impedance techniques. The charge storage mechanism of the nickel oxide-based electrodes in the alkaline electrolytes is as follows^38^

2 $${\rm{NiO}}+{{\rm{OH}}}^{-}\iff {\rm{NiOOH}}+{{\rm{e}}}^{-}$$

At the oxidation process, NiO converts to NiOOH, and in the reduction reaction, NiOOH converts to NiO in a reversible process, which is the mark of the pseudocapacitance nature of the electrode material.

#### Three electrodes measurements

**Figure 7:**
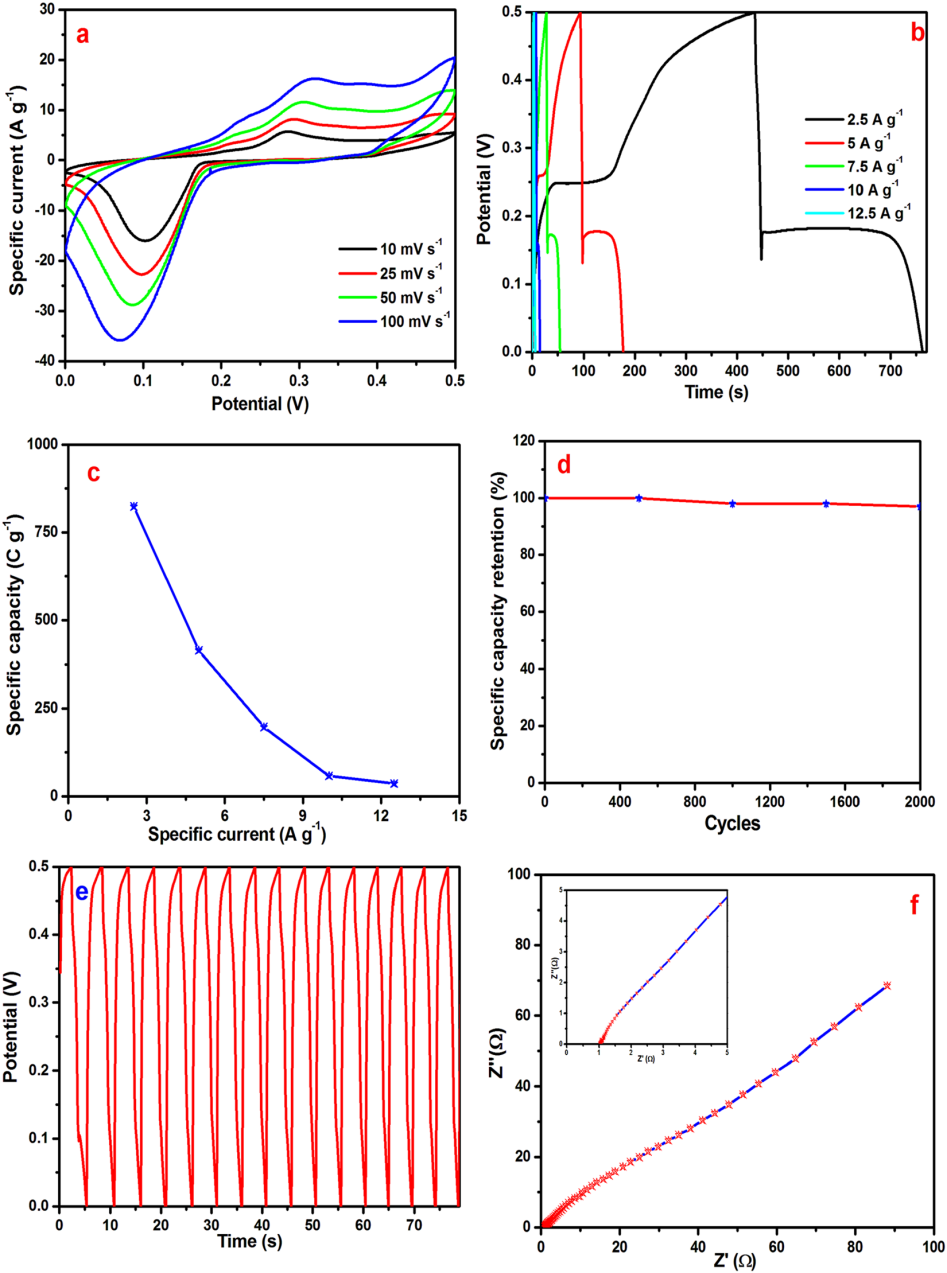
(**a**) CV curves of Ag/NiO nanoarrays electrode, (**b**) CHDH curves of Ag/NiO nanoarrays electrode (**c**) specific current against specific capacity. (**d**) Cyclic stability of Ag/NiO nanoarrays electrode, (**e**) first 25 cycles and (**f**) Nyquist plot of Ag/NiO nanoarrays electrode(inset shows the low frequency region).

CV measurements were carried out over the potential range, 0 to 0.5 V. Figure 7(a) shows the CV curves at the different scan rates, indicating the good redox behavior of the Ag/NiO honeycomb nanoarray electrode (battery type). These curves indicate that at higher scan rates, the area under the curve is high, which may be because the electrolyte ions react only with the surface of the electrode materials, whereas at lower scan rates, the area under the curve is decreased because the electrolyte reacts with the inner active sites of the Ag/NiO nanoarrays electrode (ion exchange mechanism). Figure 7(b) shows the charge-discharge curve of the Ag/NiO honeycomb nanoarrays electrode. The sudden potential increased at 0.12 to 0.17 V in the discharge curve this is attributed to the quasi- conversion reaction mechanism which has explained by the Lou’s group^39^. This reaction differs from conventional ion intercalation mechanism of electrochemistry. The similar behavior was observed for Ag/NiO nanoarrays electrode which indicates the charges stored by quasi-conversion reaction mechanism. The shape of the charge-discharge curve indicates the non-linear behavior of the Ag/NiO nanoarrays electrode, which is similar to the battery type electrode curve^39,40^. The specific capacity was estimated from the following equation due to the battery type behavior of the charge-discharge curves^38,41,42^

3 $$Cs=\frac{I{\rm{\Delta }}t}{m3.6}$$

where Cs is the specific capacity (mA h g^−1^); I is the current (A); Δt is the discharging time (s), and m is the mass of active material (g).). The calculated specific capacity was 824 C g^−1^ (229 mA h g^−1^), 415 C g^−1^ (115 mA h g^−1^), 197 C g^−1^ (55 mA h g^−1^), 58 C g^−1^ (16 mA h g^−1^), and 36 C g^−1^ (10 mA h g^−1^) at a specific applied current of 2.5, 5, 7.5, 10, and 12.5 A g^−1^, respectively. The Ag/NiO honeycomb nanoarrays delivered a maximum specific capacity of 824 C g^−1^ at a specific current of 2.5 A g^−1^.

Y. Yang *et al*.^23^ produced dilute NiO/carbon nanofiber composites synthesized from the metal-organic framework and reported a specific capacitance of 234 F g^−1^ at a scan rate of 1 mV s^−1^. The present study achieved a 3.5 times higher specific capacitance at a higher applied current rate. X. Zhang *et al*.^43^ reported the investigation of a branchlike MoO_3_/Polypyrrole hybrid with enhanced electrochemical performance used as an electrode in supercapacitors. This MoO_3_/Polypyrrole nanocomposite electrode delivered a maximum specific capacity of 123 C g^−1^ (123 F g^−1^) at 0.27 A g^−1^. Edge-enriched graphene quantum dots for enhanced photo-luminescence and supercapacitance was reported by M. Hasan *et al*.^44^. They found graphene quantum dots electrode yields the specific capacitance of 236 F g−1 at a specific current of 1 A g^−1^. A simple route was used for the synthesis of electrode materials and the Ag/NiO nanoarray electrode material demonstrated better electrochemical performance than that reported in the literature. Figure 7(c) shows the specific current as a function of the specific capacity. From this plot, the specific capacity decreases with increasing applied specific current due to the ion exchange mechanism. Long term cycling stability is an important parameter of supercapacitors, which was measured over 2000 continuous charge-discharge cycles. Figure 7(d and e) presents the specific capacity retention of the Ag/NiO nanoarrays electrode every 500 cycles and first fifteen cycles of the electrode. The results revealed 96% retention of the original specific capacity after 2000 cycles. N. Padmanathan *et al*.^24^ reported a NiO-In_2_O_3_ micro flower (3d)/nanorod hetero-architecture as the supercapattery electrode with good cycling stability; the maximum cycling capacity retention was 89.5% after 5000 cycles.

Electrochemical ac-impedance is the best tool for understanding the charge-transfer kinetics at the electrode/electrolyte interface. The Nyquist plot is presented in Fig. 7(f). The ac-impedance has measured at the open circuit potential and fitted with an equivalent circuit (fitting parameters data was given in supporting information table ST1 and fitting circuit S2) using IVAM workstation inbuilt software. This circuit consists of the solution resistance (R_s,_ which was observed between the counter and reference electrodes), charge transfer resistance (R_ct_ which is measured by the single kinetically-controlled electrochemical reactions, the electrolyte ion charge transfer has a certain speed which is mainly depends on the kind of reaction, temperature, concentration of the reaction products and the applied open circuit potential), Warburg element (ion diffusion resistance depends on the frequency of the potential perturbation. At low frequencies ion diffused very well due to the electrolyte ions have enough time to diffuse all active sites of the electrode whereas Warburg impedance is very low since the electrolyte ions do not diffuse all active sites of the electrode) Z_w_, pseudocapacitor (C_p_ which is attributed to the Faradic redox reaction) and double layer capacitor (C_dl_ double layer region exist between the electrode/surrounding electrolyte interface which is formed form the electrolyte ions adsorbs onto the electrode surface). The ac-impedance spectrum consists of two regions which are high frequency regions and low frequency regions. At the high frequency region we have observed a semicircle due to the polarization effect^45,46^. A straight line was observed at lower frequency region which is lower than that of 90° angle to the real axis due to the mass transfer effect or superposition effect. A charge transfer resistance of 1.8 Ω was obtained from the Nyquist plot. This low charge transfer resistance indicates the high conductivity of the Ag/NiO nanoarrays electrode.

#### Two electrodes measurements (Asymmetric supercapacitor device)

All the electrochemical studies show that the Ag/NiO honeycomb nanoarrays are a suitable positive electrode material for supercapacitor device fabrication. Based on the above results, an asymmetric supercapacitor device was fabricated using the mass balance equation (Please see the device fabrication details in the supporting information). The fabricated asymmetric device was examined by CV, charge-discharge, and ac-impedance measurements.

**Figure 8:**
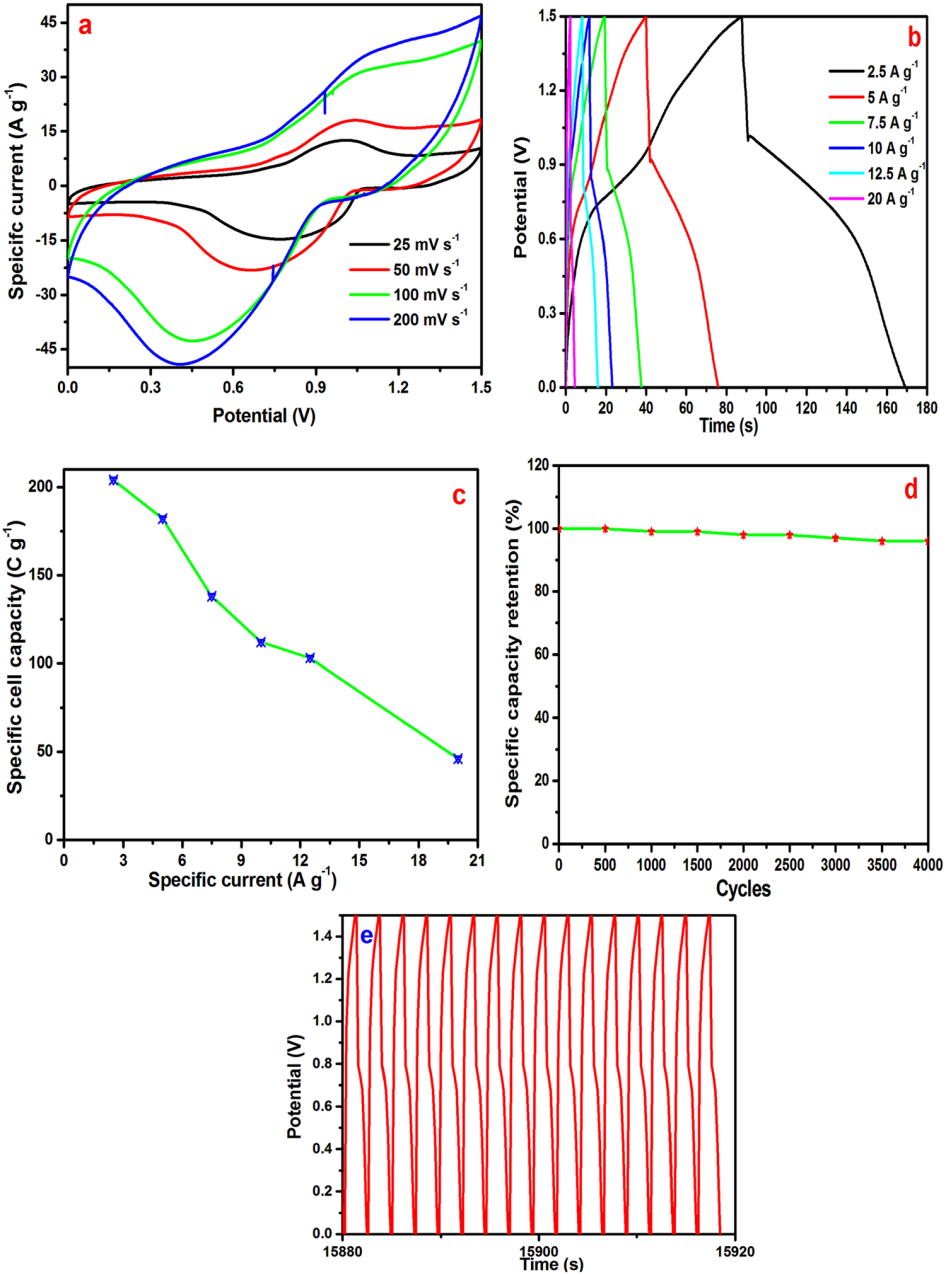
(**a**) CV curves of asymmetric device, (**b**) CHDH curves of asymmetric device, (**c**) specific current against specific cell capacity, (**d**) cyclic stability, and (**e**) last 15 cycles of asymmetric device.

Figure 8(a) shows the CV curves of the asymmetric device over the potential range of 0 to 1.5 V. The shape of the CV curves indicates the good redox behavior of the electrode. Figure 8(b) shows the charge-discharge curves. The specific capacity was estimated from equation 3. The estimated specific cell capacity was 204 C g^−1^ (56.6 mA h g^−1^), 182 C g^−1^ (50.5 mA h g^−1^), 138 C g^−1^ (38.3 mA h g^−1^), 112 C g^−1^ (38.1 mA h g^−1^), 103 C g^−1^ (28.5 mA h g^−1^), and 46 C g^−1^ (12.7 mA h g^−1^) at an applied specific current of 2.5, 5, 7.5, 10, 12.5, and 10 A g^−1^, respectively. Figure 8(c) shows the applied specific current as a function of the specific cell capacity. The 4000 continues charge-discharge cycles were measured for cycling stability analysis. The asymmetric device showed 96% capacity retention after 4000 continuous charge-discharge cycles, which are presented in Fig. 8(d and e).

**Figure 9:**
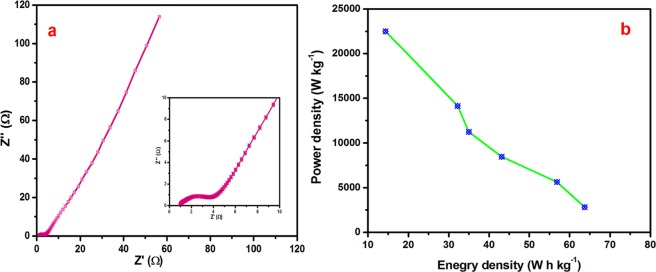
(**a**) Nyquist plot of asymmetric device (inset shows the low frequency region) and (**b**) Ragone plot of asymmetric device.

Another key factor of the supercapacitors is the energy density and power density, which were estimated from the following equations^47^

4 $$E=\frac{1}{2}C{V}^{2}$$

5 $$P=\frac{E}{t}$$

where E is the specific energy density (W h kg^−1^); C is the specific cell capacity (C g^−1^); V is the potential range (V); P is the power density(W kg^−1^), and t is the discharge time (s). The Ragone plot is shown in Fig. 9(a). The device yielded an energy density of 63.75, 56.87, 43.12, 35, 32.18 and 14.37 W h kg^−1^ corresponding to a power density of 2812.5, 5625, 8463.5, 11250, 14131.09, and 22500 W kg^−1^, respectively. K. Brousse *et al*.^48^ have reported that electrochemical behavior of high performance on-chip porous carbon films for micro-supercapacitors applications in organic electrolytes. Te device delivered the specific energy density of 210 mW h cm3 corresponding specific power of 12.6 W cm3. Advanced electrochemical energy storage supercapacitors based on the flexible carbon fiber fabric-coated with uniform coral-like MnO_2_ structured electrodes have been reported by M. Cakici *et al*.^49^. The hybrid device demonstrates the excellent specific energy density of 20 W h Kg^−1^. X. Zhou *et al*.^50^ have reported that the bamboo-like composites of V_2_O_5_/Polyindole and activated carbon cloth as electrodes for all-solid-state flexible asymmetric supercapacitors. The solid state device demonstrates the high energy density of 38.7 W h kg^−1^ at a power density of 900 W kg^−1^. In the present work Ag/NiO nanoarrys electrode yields the higher energy and power density than other reports. These results are higher than those of lead acid batteries and comparable to those of metal hydride batteries. Figure 9(b) shows the Nyquist plot of the asymmetric device. The charge transfer resistance of (Rct) 2.91 Ω was estimated from this plot. The low charge transfer resistance offered a high power performance of the asymmetric supercapacitor device.

## Conclusion

Ag/NiO nanoarrays were synthesized using a hydrothermal method. XRD and XPS confirmed the crystal structure of the Ag/NiO nanoarrays and the presence of metallic silver, respectively. The honeycomb structured nanoarrays were identified by FESEM and HRTEM. The electrochemical studies indicated that the Ag/NiO nanoarray is a suitable electrode material for supercapacitor applications. The asymmetric device was fabricated and tested, which yielded a high specific cell capacity of 204 C g^−1^ at a specific current of 2.5 A g^−1^ and a maximum energy density of 63.75 W h kg^−1^ at a power density of 2812.5 W kg^−1^. These results are higher than those of lead acid batteries and comparable to those of metal hydride batteries.

## Supplementary information

Supplementary Information
