# Supplementary material for: Synthesis of Ag/NiO Honeycomb Structured Nanoarrays as the Electrode Material for High Performance Asymmetric Supercapacitor Devices
Source: Sci Rep. 2019 Mar 19;9:4864. doi: 10.1038/s41598-019-41446-0 (PMC6424974; doi:10.1038/s41598-019-41446-0)
Supplement: Supplementary file 1 — Supplementary Information [file 41598_2019_41446_MOESM1_ESM.pdf]

SUPPLEMENTARY INFORMATION

Synthesis of Ag/NiO Honeycomb Structured Nanoarrays as the Electrode Material for High Performance Asymmetric Supercapacitor Devices

Sadayappan Nagamuthu and Kwang-Sun Ryu*

Department of Chemistry, University of Ulsan, Muger-dong, Nam-gu, Ulsan 680-749, Republic of Korea

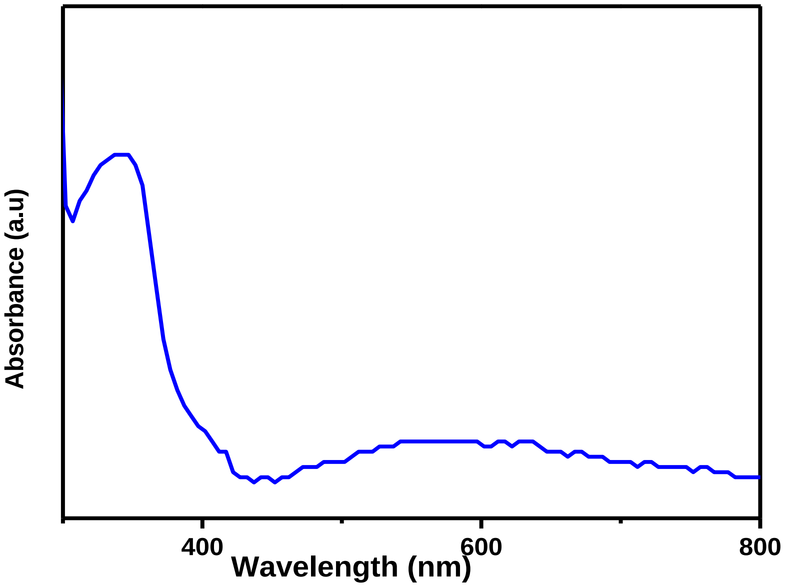

Figure S1: UV-Vis absorbance spectrum of Ag/ NiO honeycomb nanoarrys

TableST1: AC-impedance spectrum fitting parameters of equivalent circuit data of Ag/ NiO honeycomb nanoarrys electrode

	Parameter	Value	Error%	Min	Max	Unit
Par[1]	Rs	1.39E+00	0.44	1.00E-04	1.00E+13	Ohm
Par[2]	Rct	8.26E+00	1.06	1.00E-04	1.00E+13	Ohm
Par[4]	Cp	1.76E-03	0.96	1.00E-13	1.00E+01	F
Par[5]	Cdl	4.21E-02	0.85	1.00E-13	1.00E+01	F
Par[6]	W	3.11E-02	1.27	1.00E-12	1.00E+06	1/(Ohm sqrt(Hz)

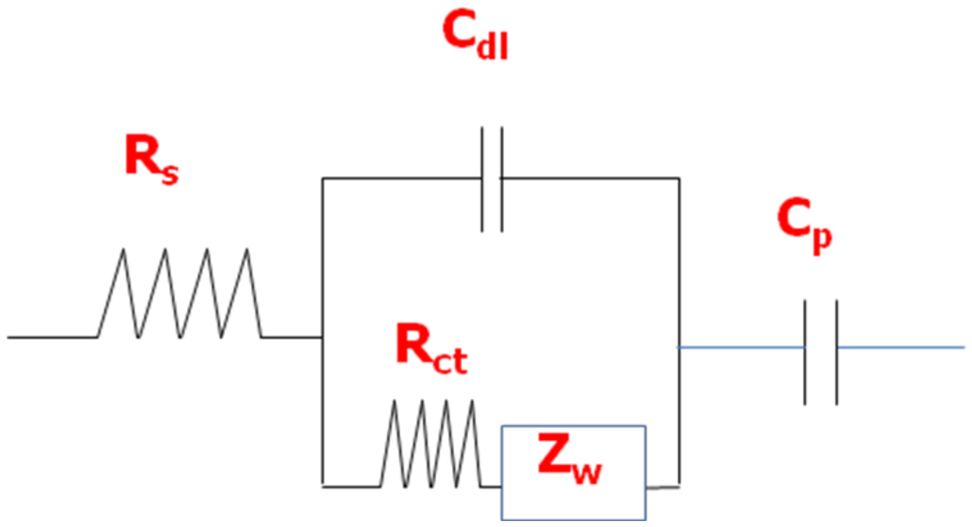

Figure S2: AC-impedance spectrum fitting parameters of equivalent circuit data of Ag/ NiO honeycomb nanoarrys electrode

Asymmetric supercapacitor device Fabrication:

A coin cell was fabricated using silver nickel oxide nanoarrays as the positive electrode (1 mg) and activated carbon as the negative electrode (5.55 mg). Total mass of the electrode material is 6. 55 mg. Whatman filter paper was used as the separator, which had been presoaked in a 2 M KOH electrolyte for 24 h prior to device fabrication.

Mass balancing for Device fabrication:

The electrode mass ratio should be optimized prior to asymmetric device fabrication

$$\frac{m_+}{m_-} = \frac{C_- \, X \, V_-}{C_+ \, X \, V_+}$$

where m_+ is mass of the positive electrode (activated carbon) (g), m_- is the mass of the negative electrode (g), C_- is the specific capacity of the negative electrode (C g^{-1}), V_- is the potential range of the negative electrode (V), C_+ is the specific capacity of the positive electrode (C g^{-1}), and V_+ is the potential range of the positive electrode (V). The optimal mass ratio is ~0.18 is the positive to negative electrodes.

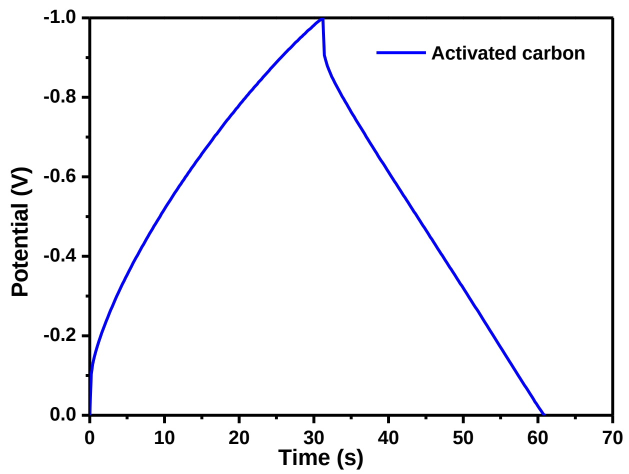

Figure S3: Charge discharge curve of activated carbon electrode at 2.5 A g^{-1} in aqueous electrolyte
